# XJB-5-131 inhibited ferroptosis in tubular epithelial cells after ischemia−reperfusion injury

**DOI:** 10.1038/s41419-020-02871-6

**Published:** 2020-08-14

**Authors:** Zhi Zhao, Jianliang Wu, Huzi Xu, Cheng Zhou, Bicui Han, Han Zhu, Zhizhi Hu, Zhimei Ma, Zhangyin Ming, Ying Yao, Rui Zeng, Gang Xu

**Affiliations:** 1 Division of Nephrology, Tongji Hospital, Tongji Medical College, Huazhong University of Science and Technology, 1095 Jiefang Ave, 430030 Wuhan, Hubei China; 2 Department of Endorenal Rheumatism, Wenchang People’s Hospital, 571300 Wenchang, Hainan Province China; 3 Wuhan Institute of Biotechnology, 430000 Wuhan, China; 4 Department of Pharmacology, School of Basic Medicine, Tongji Medical College, Huazhong University of Science and Technology, 13 Hangkong Road, 430000 Wuhan, Hubei China

**Keywords:** Acute kidney injury, Drug development

## Abstract

Regulated necrosis has been reported to exert an important role in the pathogenesis of various diseases, including renal ischemia-reperfusion (I/R) injury. Damage to renal tubular epithelial cells and subsequent cell death initiate the progression of acute kidney injury (AKI) and subsequent chronic kidney disease (CKD). We found that ferroptosis appeared in tubular epithelial cells (TECs) of various human kidney diseases and the upregulation of tubular proferroptotic gene ACSL4 was correlated with renal function in patients with acute kidney tubular injury. XJB-5-131, which showed high affinity for TECs, attenuated I/R-induced renal injury and inflammation in mice by specifically inhibiting ferroptosis rather than necroptosis and pyroptosis. Single-cell RNA sequencing (scRNA-seq) indicated that ferroptosis-related genes were mainly expressed in tubular epithelial cells after I/R injury, while few necroptosis- and pyroptosis-associated genes were identified to express in this cluster of cell. Taken together, ferroptosis plays an important role in renal tubular injury and the inhibition of ferroptosis by XJB-5-131 is a promising therapeutic strategy for protection against renal tubular cell injury in kidney diseases.

## Introduction

Renal ischemia−reperfusion injury (I/R) often occurs following infection, trauma or surgery^1,2^. However, few therapeutic interventions demonstrate promising efficacy in postponing renal I/R injury and improving patient survival. The pathophysiology of renal I/R injury is characterized by renal tubular damage, inflammation and vascular dysfunction, during which tubular cell death is considered the primary pathogenic event. Renal tubular epithelial cells (TECs) are the main cells in the kidney and are vulnerable to injuries, including toxins, hypoxia, mechanical injury and senescence. Accumulating evidence suggests that maladaptive repair of TECs has a decisive role in the progression of acute kidney injury (AKI) to chronic kidney diseases (CKD)^3–6^. As tubular injury are the precipitating factors in AKI, understanding the mechanism of tubular cell death is important for fundamentally treating ischemic kidney injury. Multiple forms of cell death have been indicated to be involved in ischemic injury; however, the optimal treatment strategy is still unclear.

Regulated necrosis, which is characterized by cell swelling and destruction of the integrity of the cellular membrane, has been considered to be the dominant contributor to acute tubular necrosis, including pyroptosis, necroptosis and ferroptosis. Pyroptosis is a programmed cell death that is characterized by the release of cellular contents and activation of a strong inflammatory response that relies on the enzymatic activity of inflammatory proteases with the regulation of caspases and gasdermin D (GSDMD)^7–10^. Necroptosis is a form of regulated necrosis that is mediated by the proteins RIPK3 and MLKL^11–13^. Necroptosis and pyroptosis have been well studied in kidney diseases. However, ferroptosis attracted attention just since its first definition in 2012 ^14^. Ferroptosis is characterized by the occurrence and accumulation of intense lipid peroxidation. Toxic lipid hydroperoxides (L-OOH) are converted into nontoxic lipid alcohols (L-OH) by glutathione peroxidase 4 (GPX4), which is the key enzyme that suppresses ferroptosis^15^. Ferroptosis is a complicated and intricate biological process involving amino acid, iron, and polyunsaturated fatty acid metabolism and the biosynthesis of glutathione and coenzyme Q10 ^16,17^. It has been confirmed that ferroptosis is the main death pathway rather than necroptosis to tubular cell death in mice AKI^18,19^; however, there is less evidence of ferroptosis in human kidney diseases, lack of comparison among necroptosis, ferroptosis and pyroptosis, and short of a defined treatment strategy.

XJB-5-131 is a mitochondrial-targeted nitroxide with a dual antioxidant effect that contains the radical scavenger TEMPO and the mitochondria-targeted semi-gramicidin S^20,21^, which has been shown to restore mitochondrial function, suppress DNA damage and inhibit apoptosis to protect against Huntington’s disease^20,22,23^, hemorrhagic shock^24^ and cardiac ischemia−reperfusion injury^25^. In addition, a previous study provided novel evidence that XJB-5-131 inhibits ferroptosis by suppressing lipid peroxidation in vitro^26^. However, the role of XJB-5-131 in kidney ischemic injury has not been identified yet.

The emergence of single-cell RNA sequencing (scRNA-seq) has provided a novel approach to investigate regulated necrosis in kidney diseases^27,28^. We performed scRNA-seq and focused on regulated necrosis-related genes in renal cells after I/R injury. In this study, we found that (i) the expression of ACSL4 in TECs was associated with kidney function; (ii) the genes involved in ferroptosis were most regulated in TECs; (iii) XJB-5-131 showed high affinity for TECs and inhibited I/R-induced ferroptosis in TECs and improved ischemic renal injury. Taken together, we confirmed the critical role of ferroptosis in TECs and provided a kidney-specific ferroptosis inhibitor for kidney diseases.

## Results

### The expression of proferroptotic gene ACSL4 in TECs was associated with renal function in human acute tubular injury

**Fig. 1:**
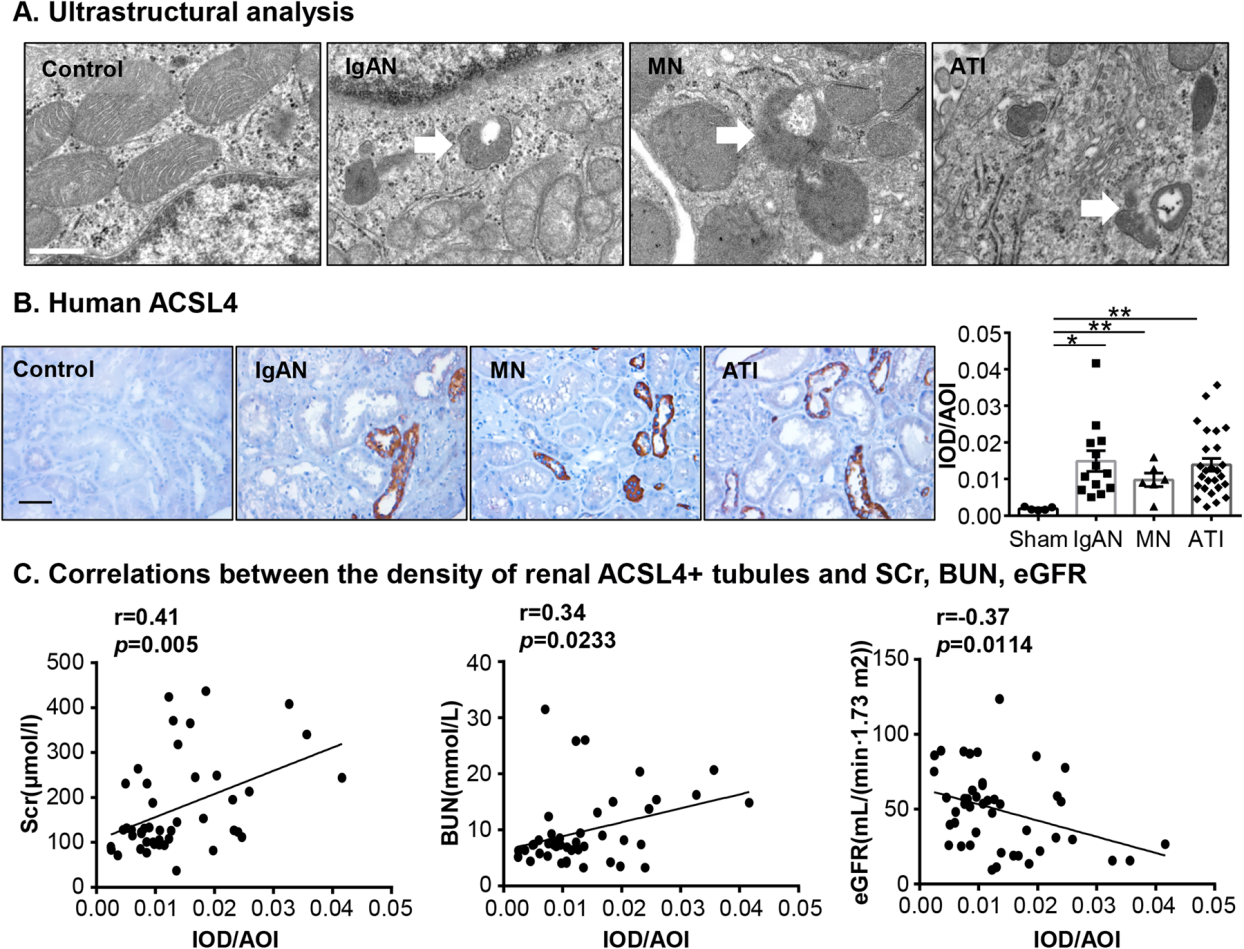
Proferroptotic gene ACSL4 TEC ferroptosis-related genes were increased in human kidney diseases. **a** Transmission electron microscopy of human tubular epithelial cells with kidney diseases, including IgA nephropathy (IgAN), membranous glomerulonephritis (MN), and acute tubular injury (ATI). The white arrowheads indicated shrunken mitochondria with outer membrane rupture. Scale bars = 500 nm. **b** The representative image and graph of ACSL4 immunostaining in TECs in human kidney diseases. **c** Correlations between the integrated option density (IOD) of intrarenal ACSL4 + immunostaining and SCr, BUN, eGFR in ATI patients. AOI area of interesting. Original magnification ×400. Scale bars = 50 μm. Data were presented as the means ± SEM. *p < 0.05, **p < 0.01.

By using transmission electron microscopy, we observed the mitochondria damage in human TECs with IgA nephropathy (IgAN), membranous glomerulonephritis (MN) and acute tubular injury (ATI). The mitochondria in injured tubular cells turned to be smaller in size with outer membrane rupture which was the distinctive morphological feature compared to other forms of regulated necrosis^14,29^ (Fig. 1a). Since acyl-CoA synthetase long-chain family member 4 (ACSL4) was identified as a mediator in ferroptosis across multiple cell types^29–31^, we detected the ACSL4 expression in human kidney diseases. Increased expression of ACSL4 was found in renal biopsy specimens of IgAN, MN and acute tubular injury patients (Fig. 1b). Next, we stained ACSL4 in renal biopsy samples from patients with acute tubular injury to further explore the relationship between ACSL4 expression and human tubular injury. As shown in Fig. 1c, a significant correlation was found between the density of ACSL4+ signal in human renal biopsies and serum creatinine (*r* = 0.41, *p* < 0.05), blood urea nitrogen (*r* = 0.34, *p* < 0.05) and eGFR (*r* = −0.34, *p* < 0.05). To further define the relationship between ACSL4 and other clinical manifestations, the patients were divided into two groups according to the ACSL4 expression in kidney biopsies: the ACSL4^high^ group (15 cases, with the highest tercile ACSL4 IOD/AOI) and the ACSL4^low^ group (30 cases, with the second lowest tercile ACSL4 IOD/AOI). Patients in the ACSL4^high^ group had increased levels of serum creatinine and blood urea nitrogen and decreased eGFR at the time of biopsy (Table 1). However, less differences were present in hematuria, proteinuria, hemoglobin (Hb), serum uric acid, albumin or total cholesterol between these two groups. Overall, our data indicate that ferroptosis was a pathological risk factor for human kidney diseases.

**Table 1:** ATI patients in the ACSL4^high^ group showed a decline of kidney function.

Variables	All (*n* = 45)	ACSL4^low^ (*n* = 30)	ACSL4^high^ (*n* = 15)	*P*
Gender				
Male	25	15 (50%)	10 (66.7%)	
Female	20	15 (50%)	5 (33.3%)	0.2888
Age (years)	46.0 (27.5, 54.5)	50.0 (34.0, 57.25)	33.0 (26.0, 52.0)	0.0495
Hematuria				
<3+ (%)	21	19 (70.4%)	12 (84.6%)	
≥3+ (%)	14	11 (29.6%)	3 (15.4%)	0.2549
Proteinuria				
<3+ (%)	25	18 (60.0%)	7 (46.7%)	
≥3+ (%)	20	12 (40.0%)	8 (53.3%)	0.3961
Hb(g/L)	122.0 (107.0,135.0)	123.0 (114.8,136.8)	114.5 (98.93,130.1)	0.2533
Blood urea nitrogen (mmol/L)	7.4 (6.1, 12.8)	7.2 (5.7, 8.1)	13.8 (7.4, 16.3)	0.0151
Serum creatinine (μmol/L)	128.0 (100.5, 237.5)	121.5 (94.8, 136.0)	244.0 (127.0, 340.0)	0.0026
Serum uric acid (μmol/L)	383.1 (324.8, 423.5)	377.2 (324.0, 416.2)	415 (337.0, 417.0)	0.1980
eGFR (mL/min/1.73 m^2^)	51.5 (25.9, 58.8)	56.2 (40.6, 69.4)	26.7 (18.9, 55.0)	0.0084
Serum albumin (g/L)	37.2 (25.7, 42.7)	37.7 (30.2, 44.7)	34.4 (18.1, 40.8)	0.1288
Serum total cholesterol	4.7 (4.0, 7.5)	4.6 (3.8, 5.9)	5.0 (4.0, 9.1)	0.2485

Data are presented as a percentage or median (25th−75th percentiles); eGFR (ml/min/1.73 m^2^) = 186 × Scr − 1.154 × age − 0.203 × 0.742 (if female) × 1.227; comparisons between groups were performed by chi-squared tests.

*Hb* hemoglobin, *eGFR* estimated glomerular filtration rate.

### XJB-5-131 treatment attenuated kidney injury and promoted TEC repair after I/R injury

**Fig. 2:**
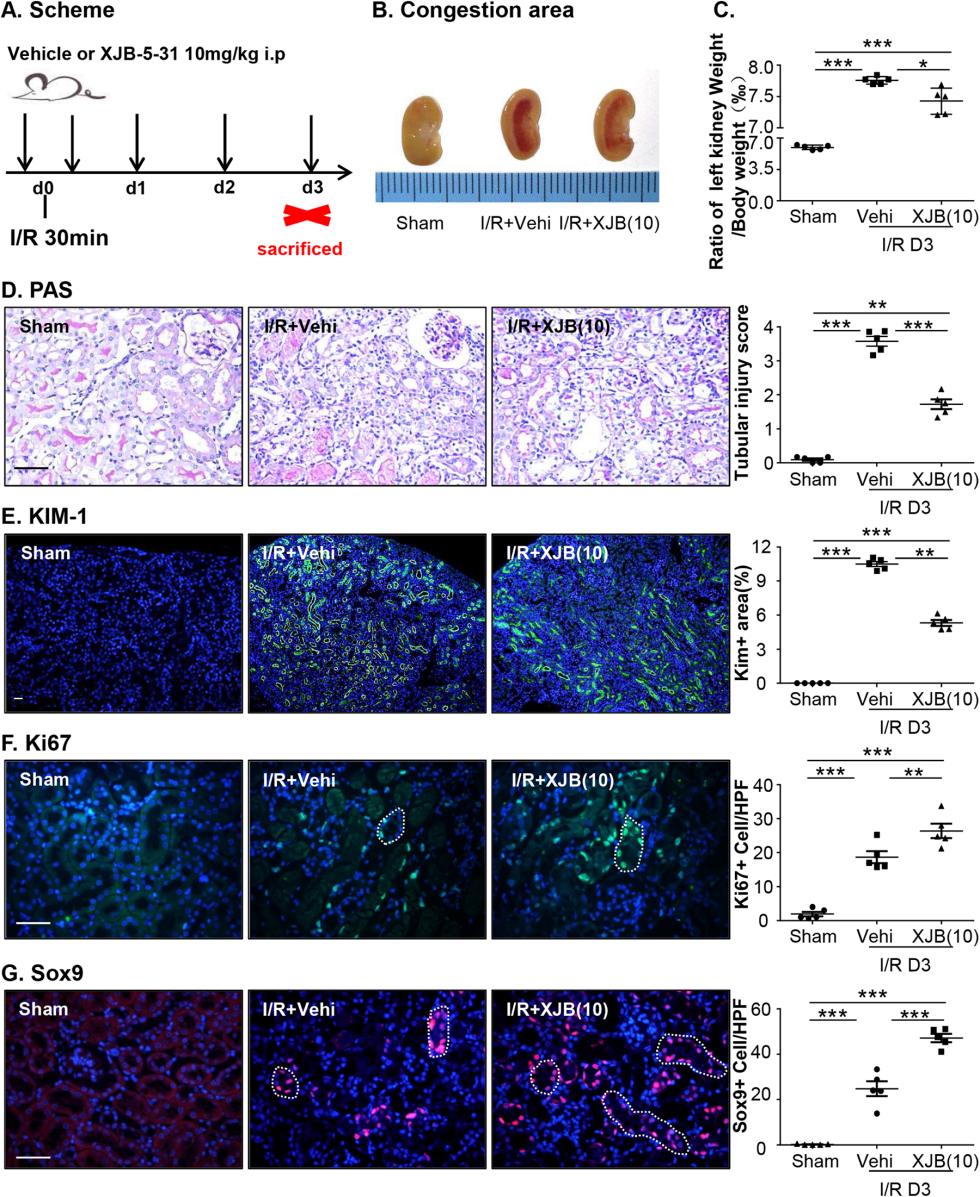
XJB-5-131 administration attenuated kidney ischemic injury in mice. **a** Scheme of the experiment. **b** Gross appearance on congestion area of representative kidneys from each group. **c** Analysis of the ratio of the left kidney to body weight. **d** XJB-5-131 decreased histologic injury in kidneys from mice with unilateral I/R at day 3. Representative image of periodic acid-Schiff (PAS) and tubular damage was scored semiquantitatively in PAS staining. Original magnification ×400. **e** The representative image of immunofluorescence staining of KIM-1 in kidney sections. Original magnification ×100. Representative image of Ki67 (**f**) and Sox9 (**g**) staining showed the proliferation in TECs after I/R injury at day 3. The number of Ki67- and Sox9-positive nuclei was measured in 6−8 visual fields. Original magnification ×400. *N* = 5/group. Scale bars = 50 μm. Data were presented as the means ± SEM. *p < 0.05, **p < 0.01,***p < 0.001.

XJB-5-131 has shown beneficial effects in degenerative diseases and anti-ferroptotic effects in HT-1080 cells^26^, but has never been studied in kidney diseases. To investigate the effect of XJB-5-131 in mice after renal I/R injury, a 10 mg/kg dose of XJB-5-131 was injected into the mice based on the experimental data in Supplementary Fig. 1. We found an obvious congestion area at the junction of the cortex and medulla in injured kidneys after I/R injury at day 3, which was reduced in the XJB treatment group (Fig. 2b). When calculating the ratio of injured kidney weight to body weight, the I/R group had a larger volume of kidney than that of the sham group but the volume decreased after XJB-5-131 treatment (Fig. 2c).

Then, we performed pathological analysis of tubular injury to assess whether XJB treatment improved I/R injury in the kidney. The loss of tubules was alleviated by XJB treatment (Fig. 2d). Consistent with this, the expression of KIM-1 (kidney injury molecule 1, a key mediator of AKI) in immunofluorescent staining (Fig. 2e) was decreased after XJB-5-131 treatment. As the regeneration and repair of renal TECs is critical for ischemic kidneys^32^, we detected proliferation and repair in injured renal TECs by Ki67 and Sox9 immunostaining. Sox9 promotes the intrinsic repair process in injured renal TECs^33^. The number of Ki67-positive nuclei in TECs was higher in I/R mice than in sham mice, while a much higher number was detected after XJB-5-131 treatment (Fig. 2f), and Sox9 was dramatically activated in TECs after XJB-5-131 injection compared to that of the I/R group (Fig. 2g). In summary, these data suggest that XJB-5-131 treatment alleviated kidney injury and improved regeneration and repair of injured renal TECs after I/R injury.

### XJB-5-131 treatment reduced intrarenal infiltration of inflammatory cells

**Fig. 3:**
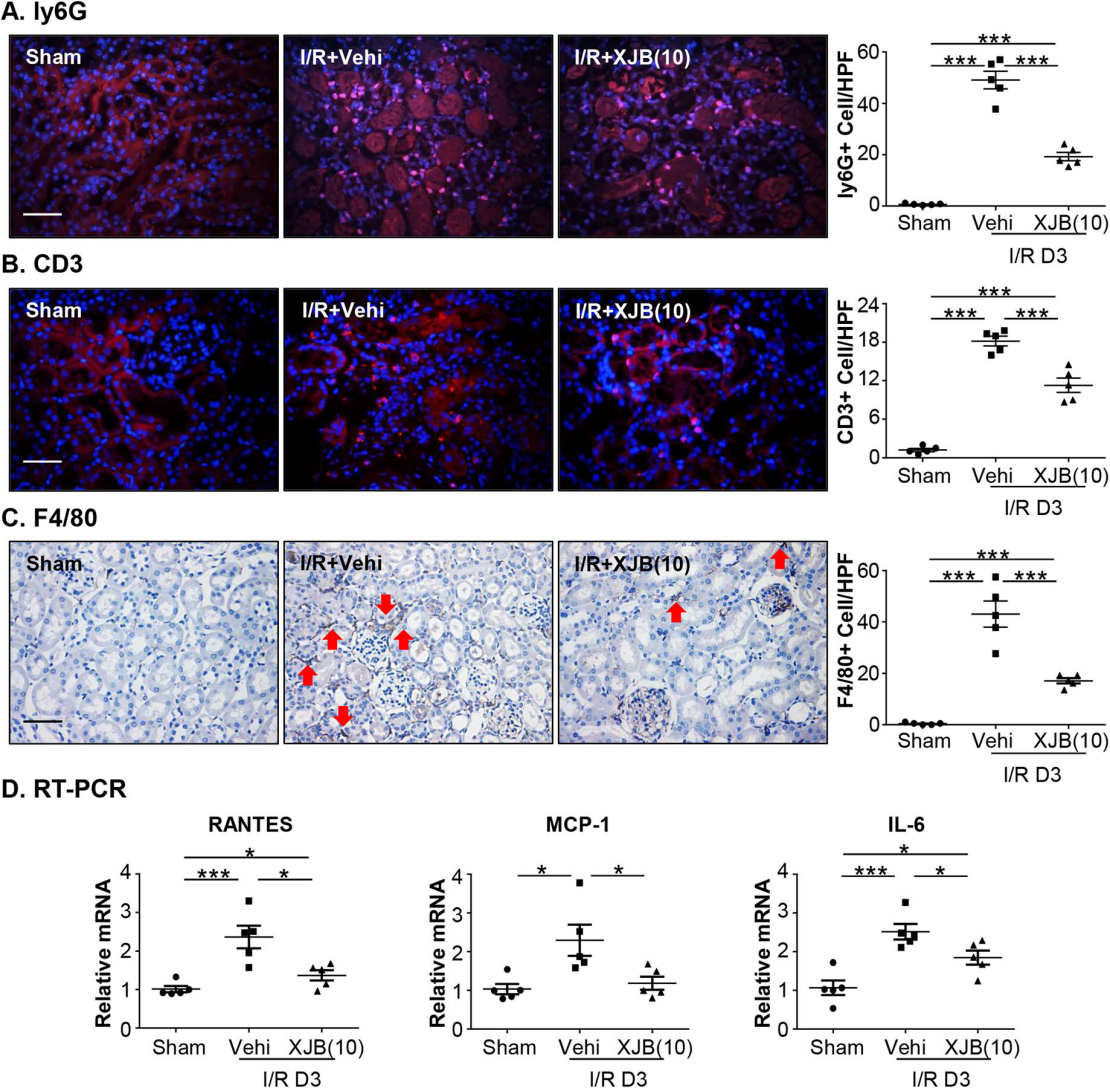
XJB-5-131 treatment reduced intrarenal infiltration of inflammatory cells triggered by ferroptosis in renal I/R. Kidney sections were labeled with antibodies against ly6G (**a**), CD3 (**b**) and F4/80 (**c**). The number of neutrophils, T cells and macrophages infiltrated into kidneys was calculated in 6−8 visual fields. The arrowheads indicated representative macrophages. **d** The expression of RANTES, MCP-1 and IL-6 mRNA in the kidneys was evaluated. *N* = 5/group. Scale bars = 50 μm. Data were presented as the means ± SEM. *p < 0.05, ***p < 0.001.

Inflammatory response has been involved in the pathogenesis of renal I/R injury^34,35^. Recent studies have demonstrated that local inflammation triggered by ferroptosis amplifies renal injury^19^. As the recruitment of neutrophils, T cells and macrophages into the site of injury participates in early inflammatory responses in AKI^36^, ly6G-, CD3- and F4/80-positive cells were calculated in kidney sections. Neutrophils, T cells and macrophages increased rapidly in the injured kidney after I/R surgery. However, in the XJB-treatment group, the infiltration of inflammatory cells in the kidney was reduced (Fig. 3a–c). Consistent with this, the expression of proinflammatory cytokines, such as RANTES, MCP-1 and IL-6, was suppressed with XJB-5-131 treatment compared to that of the I/R group (Fig. 3d).

### XJB-5-131 reduced the ferroptosis in TECs after I/R-incited kidney injury

**Fig. 4:**
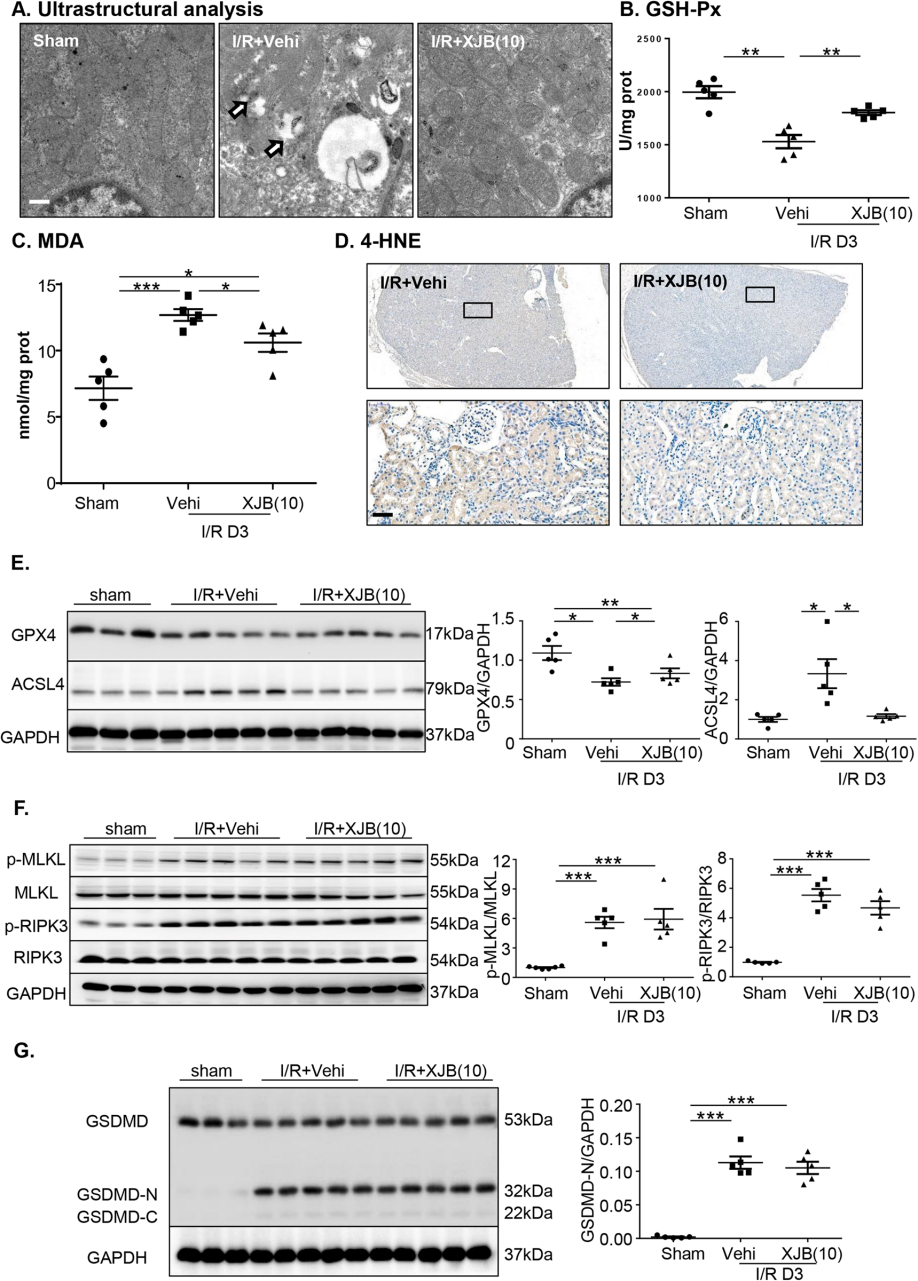
XJB-5-131 reduced the ferroptosis in TECs after I/R incited kidney injury. **a** Ultrastructural analysis revealed that upon XJB-5-131 treatment showed less outer membrane rupture (white arrows). Scale bars = 500 nm. GSH-Px (**b**) and MDA (**c**) assay showed XJB-5-131 reduced the accumulation of lipid peroxidation in kidney after ischemia injury at day 3. **d** 4-Hydroxynonenal (4-HNE) staining in mice with I/R. Scale bars = 50 μm. **e** Western blot and quantification of kidney GPX4 and ACSL4 protein. **f** The expression of RIPK3 and MLKL was assessed by immunoblotting. **g** Protein expression of GSDMD was determined by western blot. *N* = 5/group. Data were presented as the means ± SEM. *p < 0.05, **p < 0.01, ***p < 0.001.

XJB-5-131 is a potential candidate ferroptosis inhibitor in vivo^16,26^, but the impact of XJB-5-131 on I/R-induced ferroptotic cell death has not been defined. Thus, we analyzed the mitochondrial morphology and lipid peroxidation, which was identified as the execution of ferroptosis^14,37^. Electron microscopy studies revealed an increased resistance of mitochondria in TECs to I/R injury by XJB-5-131 treatment (Fig. 4a). Next, we estimated the levels of lipid peroxidation, which is a hallmark of ferroptosis^29^ by using glutathione peroxidase (GSH-Px) and malondialdehyde (MDA) assay and 4-hydroxynonenal (4-HNE) staining. As shown in Fig. 4b, c, increased levels of GSH-Px and decreased levels of MDA were observed in the XJB-5-131 group. 4-HNE staining also showed less accumulation of lipid peroxidation after XJB-5-131 treatment (Fig. 4d), while XJB-5-131 inhibited the generation of renal MDA in I/R mice.

In addition, GPX4 and ACSL4, two vital enzymes in regulating ferroptosis^16^, were measured by immunoblotting. As shown in Fig. 4e, renal GPX4 expression decreased after ischemic injury; however, XJB-5-131 administration significantly improved GPX4 expression. In addition, Western blot analysis showed that I/R induced an obvious increase in ACSL4 expression, which was inhibited by XJB-5-131 treatment. Considering that ferroptosis and necroptosis are the two major contributors to kidney I/R injury, we also detected the protein expression of necroptosis-associated molecules receptor-interacting protein kinase 3 (RIPK3) and mixed lineage kinase domain-like (MLKL)^38,39^, but there was no difference in the expression of phosphorylated RIPK3 or phosphorylated MLKL between the two groups with or without XJB-5-131 administration (Fig. 4f). Besides necroptosis and ferroptosis, the role of another form of regulated necrosis, pyroptosis, has also been confirmed in I/R kidney^40,41^. Therefore, we detected the level of GSDMD-N that was involved in the formation of a plasma membrane pore to trigger pyrotosis. As shown in Fig. 4g, I/R insult significantly increased the cleavage of GSDMD, but no statistically significant difference was observed in the two groups with XJB-5-131 treatment or not. In conclusion, XJB-5-131 protected injured kidneys from I/R-induced ferroptosis but not necroptosis and pyroptosis.

### XJB-5-131 presented superior plasma stability, fast plasma-kidney transfer and high renal affinity in mice

**Fig. 5:**
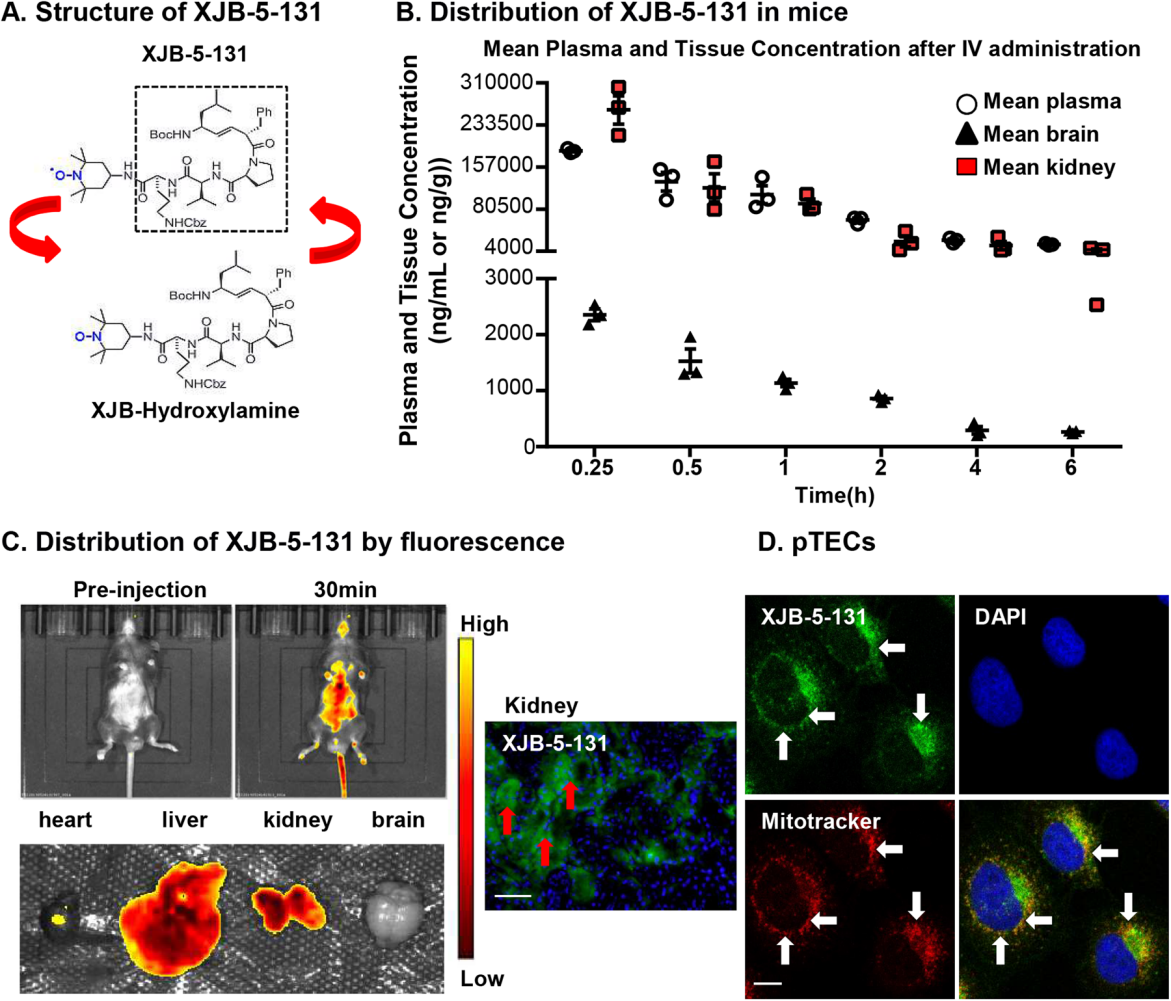
Pharmacokinetics and distribution analysis of XJB-5-131 in vivo and in vitro. **a** The structure of XJB-5-131. **b** The tissue concentrations of XJB-5-131 in mice. Mice were injected with XJB-5-131 and sacrificed after 0.25, 0.5, 1, 2, 4 and 6 h. Plasma, kidney, liver and brain were collected for in vivo-based pharmacokinetic assays by LC-MS/MS. XJB-5-131 is unstable in liver homogenates, and so the data could not be obtained. *N* = 3/group. Data are presented as the means ± SEM. **c** Mice were intravenously injected with BODIPY-FL-labeled XJB-5-131. Whole-body fluorescence imaging was performed at 0 and 30 min, and the fluorescence intensity of various organs (heart, kidney, liver and brain) was measured after 30 min. Frozen sections were used to visualize the distribution of XJB-5-131 in the kidney. The red arrows show representative TECs stained by XJB-5-131. Scale bars = 50 μm. Original magnification ×400. **d** XJB-5-131 targeted mitochondria in pTECs. Mitochondria are labeled by MitoTracker (Red). XJB-5-131 are stained in green. The white arrowheads indicate representative mitochondria stained with XJB-5-131 and MitoTracker in pTECs. Original magnification ×600. Scale bars = 10 μm.

Since the first-generation ferroptosis inhibitor ferrostatin-1 (Fer-1) failed to achieve high plasma stability, to verify whether XJB-5-131 could be utilized for the treatment of kidney diseases, an in vivo-based pharmacokinetic model was used, and LC-MS/MS was used to analyze the drug distribution in serum and extracts from the kidney, brain and liver. The mice were sacrificed at 0.25, 0.5, 1, 2, 4 and 6 h after i.v. bolus administration of XJB-5-131. XJB-5-131 was easily converted into XJB-hydroxylamine in situ^42^; thus, the PK data obtained by LC-MS/MS were mostly from the bioanalysis of XJB-hydroxylamine. As shown in Fig. 5b and Table 2, the calculated maximal concentration (*C*_max_) of XJB-5-131 in plasma, kidney and brain occurred 15 min after vein injection at concentrations of 186.7 µg/ml, 261.1 µg/g and 235.3 µg/g, respectively. The concentration of XJB-5-131 in the liver was not obtained. The half-life (*t*_1/2_) was approximately 2 h. The area under the concentration–time curve (AUC_0−6 h_) of plasma was 322.7 µg·h/ml. The AUC_0−6 h_ in the kidney was approximately fourfold compared with that of the AUC_0−6 h_ in the brain (234.7 µg·h/g versus 4.1 µg·h/g). In general, XJB-5-131 showed fast plasma-tissue drug transfer. Moreover, the concentration in the kidney was almost close to the plasma concentration and lasted much longer, which predicted the therapeutic value of XJB-5-131 in kidney diseases. Next, we intravenously injected 3 mg/kg BODIPY-FL-XJB-5-131 into mice to explore the distribution of XJB-5-131 in the kidney, and whole-body fluorescence was analyzed at 0 and 30 min after injection. Compared with low signals in the heart and brain, higher radiant efficiency was observed in the kidney and liver. As XJB-5-131 was not detected in liver homogenate, the increased radiant efficiency in the liver indicated that XJB-5-131 was unstable in liver homogenate.

**Table 2:** PK parameters of XJB-5-131 in tissues.

PK parameters	Unit	Plasma	Brain	Kidney
*C*_max_	ng/mL or ng/g	186,667	2353	261,067
*t*_max_	Hour	0.25	0.25	0.25
AUC_0–6 h_	(ng·h/mL) or(ng·h/g)	322,745	4052	234,683
AUC_0–∞_	(ng·h/mL) or(ng·h/g)	364,783	4859	255,150
*t*_1/2_	Hour	1.77	2.07	2.23
MRT_0–∞_	Hour	1.88	1.98	1.42
MRT_0-6h_	Hour	2.65	3.14	2.05

*PK* pharmacokinetic, *C*_*max*_ maximum plasma concentration, *t*_*max*_ time to maximum plasma concentration, *AUC* area under the concentration−time curve, *t*_1/2_ biological half-life, *MRT* mean residence time.

Fluorescence microscopy indicated that the majority of XJB-5-131-positive cells were kidney TECs, which provided evidence of the high affinity of XJB-5-131 for TECs (Fig. 5c). Furthermore, XJB-5-131 is a mitochondrial-targeted nitroxide, while mitochondrial injury is one of the features of ferroptosis. Thus, we isolated primary tubular epithelial cells (pTECs) from mice and treated them with BODIPY-FL-XJB-5-131 to validate the localization of XJB-5-131 in pTECs. We found that XJB-5-131 accumulated in the cytoplasm and co-stained with mitochondria labeled by MitoTracker Red in pTECs (Fig. 5d). These data suggest that XJB-5-131 is a valuable ferroptosis inhibitor for kidney diseases, with superior plasma stability, fast plasma-kidney transfer and high affinity for renal TECs.

### Ferroptosis-related genes expressed mainly in TECs after I/R injury

**Fig. 6:**
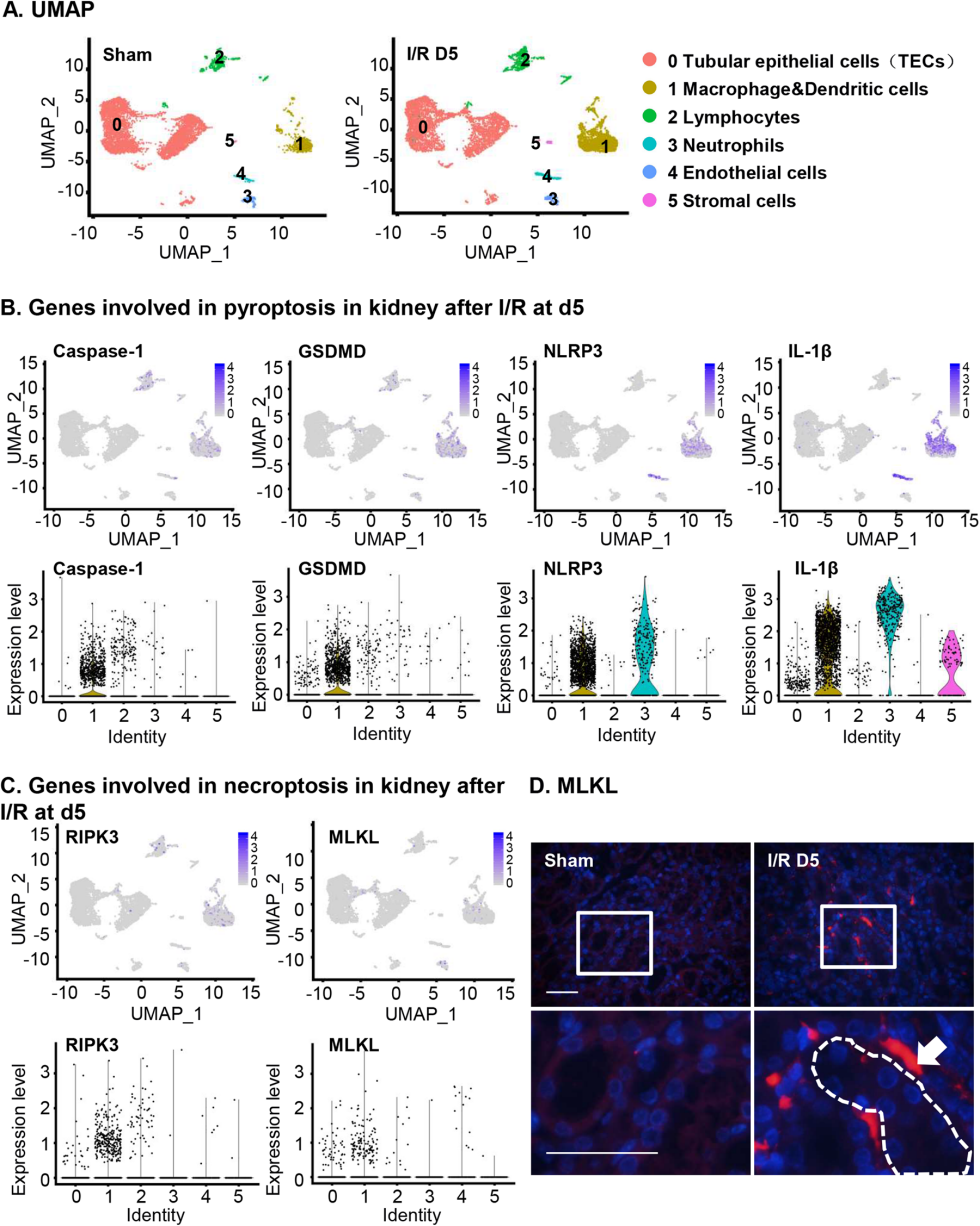
Pyroptosis- and necroptosis-related genes expressed a small amount in renal tubular epithelial cells (TECs) by scRNA-seq analysis. **a** scRNAseq identified clusters of cells in the sham and I/R kidneys after surgery at day 5. UMAP plot representation of 13,650 kidney cells, including 7581 cells from I/R kidney and 6069 cells from sham kidney. UMAP projections and accompanying violin plots depicted genes related to pyroptosis (**b**) and necroptosis (**c**) in different clusters after I/R at day 5. **d** Representative sections of MLKL immunofluorescence staining from mice kidneys after unilateral I/R at day 5. The white arrowheads indicated representative MLKL distribution in renal interstitial cells, not in renal tubules. Original magnification ×400. Scale bars = 50 μm.

**Fig. 7:**
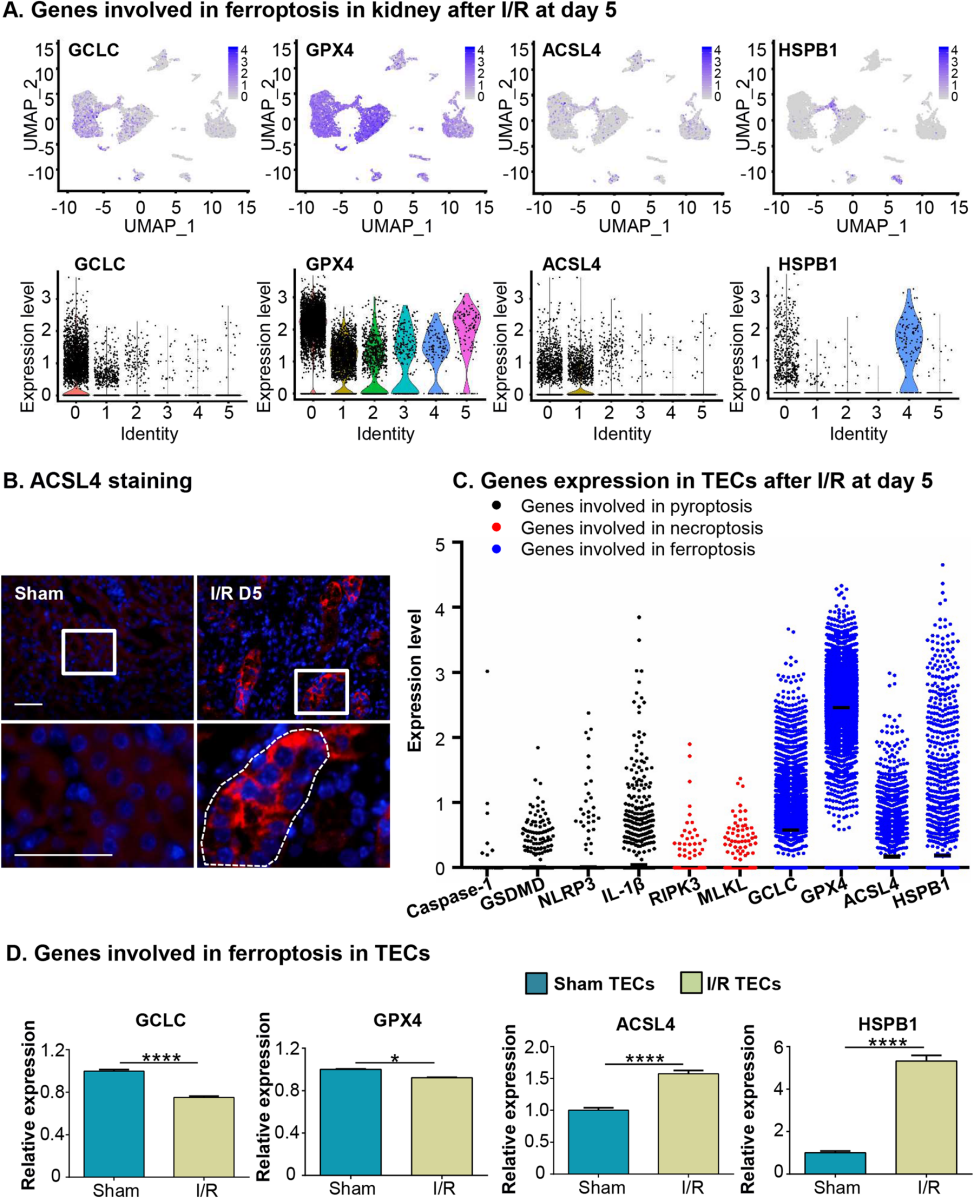
Ferroptosis-related genes expressed mainly in TECs after I/R. **a** UMAP projections and accompanying violin plots depicted genes related to ferroptosis in different clusters from kidney after I/R at day 5. **b** The representative image of immunofluorescence staining of ACSL4 in renal tubules. Original magnification ×400. Scale bars = 50 μm. **c** The expression of genes involved in different regulated necrosis pathway in TECs. **d** Box plot showed the different expression of ferroptosis-related genes between sham and I/R TECs. *p < 0.05, ****p < 0.0001.

In order to better understand the role of ferroptosis and other forms of regulated necrosis in different kidney cells, we performed single-cell sequencing in I/R kidney. Kidney specimens from sham and I/R mice were collected and digested into single cells. A total of 13,650 cells were qualified and further analyzed (Supplementary Fig. 2A), including 7581 cells from the I/R sample and 6069 cells from the sham sample. Six distinct cell populations, including TECs, macrophages, lymphocytes, neutrophils, endothelial cells and stromal cells, were identified by using canonical markers derived from published studies and online databases (Fig. 6a and Supplementary Fig. 2B). Next, we analyzed the expression of genes involved in the regulated necrosis, including pyroptosis, necroptosis and ferroptosis, in I/R kidneys, especially in TECs. As shown in Fig. 6b, pyroptosis-associated genes, including caspase-1, gasdermin D (GSDMD), NLRP3 and IL-1β, were primarily expressed in macrophages, lymphocytes and neutrophils. However, few TECs expressed these genes after I/R. GSDMD was highly expressed in renal interstitial cells than in renal tubules by immunofluorescence staining (Supplementary Fig. 4). Moreover, the necroptotic genes RIPK3 and MLKL were also specifically expressed in macrophages after I/R (Fig. 6c). Immunostaining also showed MLKL mostly distributed in renal interstitial tissue but not in TECs after I/R injury (Fig. 6d). However, the majority of TECs after I/R injury expressed ferroptosis-related genes, including glutamate-cysteine ligase, catalytic subunit (GCLC), which is involved in glutathione synthesis^15^, and GPX4, ACSL4 and heat shock protein beta 1 (HSPB1), which regulates iron metabolism, serving as an inducer and mediator of ferroptosis^16^^,^^29,43,44^ (Fig. 7a). Proferroptotic gene ACSL4 was confirmed to express in renal tubules by immunostaining (Fig. 7b). As shown in Fig. 7c, the number of TECs expressing ferroptosis-related genes was much more than that of TECs expressing the other two regulated necrosis-related genes. In addition, the expression levels of ferroptosis-associated genes were much higher than those of pyroptosis- or necroptosis-related genes. Next, we compared ferroptosis-related genes in TECs from I/R kidneys with TECs from sham-operated kidneys. The anti-ferroptotic genes GCLC and GPX4 decreased, while the proferroptotic genes ACSL4 and HSPB1 increased in TECs after I/R which suggests the important role of TECs ferroptosis in the progression of ischemic injury (Fig. 7d).

## Discussion

In this study, we first confirmed ferroptosis in the human TECs with clinical kidney diseases by transmission electron microscopy and found the expression of the proferroptotic gene ACSL4 in tubular cells was correlated with severity of kidney function decline, and we also identified that a potential ferroptosis inhibitor, XJB-5-131, which has a high affinity for the kidney, especially renal TECs, showed the potential to alleviate kidney injury, promote proliferation and repair of TECs, decrease kidney inflammation and inhibited the accumulation of lipid peroxidation but did not affect necroptosis and pyroptosis in I/R model. Thus, XJB-5-131 may be a clinical candidate for the treatment of ischemic kidney injury. Next, we performed scRNA-seq to further investigate the role of different forms of regulated necrosis in I/R kidneys, especially the TECs which are the most sensitive to the injury. We found that ferroptosis-associated genes were shown mainly in the cluster of TECs and the expression levels of these genes in TECs were higher suggesting an important role of ferroptosis in kidney diseases.

Regulated necrosis has been well-studied in kidney diseases^45,46^. The role of TECs in the progression of kidney diseases has also been identified^3–6^. In the past decades, studies have focused on the investigation of different pathway of regulated necrosis in the pathogensis of kidney diseases^18,31,40,47^. Necroptosis, pyroptosis and ferroptosis were proposed to play an important role of kidney injury. However, ferroptosis has been regarded as the main tubular cell death pathway in ischemia−reperfusion injury^18^ and folic-acid-induced kidney injury^19^. But less evidence has been shown for the ferroptotic cell death in human kidney diseases. Shrunken and damaged mitochondria, which is the unique morphological characteristics of ferroptosis, was detected by electron microscopy in human TECs with different kidney diseases in our study. Furthermore, we observed the increasing of ACSL4 expression in human kidney diseases which was also confirmed in human kidney transplants by Muller et al.^31^. In addition, we further identified the association of tubular ACSL4 expression and the severity of kidney function decline in patients with acute tubular injury. Since the importance of ferroptosis in TECs has been identified, ferroptosis inhibition might be an effective therapeutic approach to protect the kidney against I/R injury. However, as the stability of Fer-1 in plasma is poor, a more stable ferroptosis inhibitor is in need. XJB-5-131 has been reported to exert protective effects in age-related diseases^42,48,49^, Huntington’s disease^20,22,23^, penetration injury^50^, acute lung injury^51^, and hemorrhagic shock^24^, and has been confirmed to inhibit ferroptosis in vitro. We identified that XJB-5-131 attenuated kidney injury, promoted TEC proliferation and decreased inflammation after I/R. Previous study showed that XJB-5-131 suppressed lipid peroxidation in acute lung injury^51^ which was consistent with our data in the renal I/R. Moreover, we found that XJB-5-131 was stable in plasma and kidney, especially in the mitochondria of TECs. These results suggested that XJB-5-131 might be a promising therapeutic compound for tubular injury diseases.

Linkermann and colleagues have compared two forms of regulated necrosis, ferroptosis and necroptosis in I/R model but not to mention the pyroptosis. Thus we performed ScRNA-seq to compare these three pathways of regulated necrosis in different renal cells simultaneously. The development of scRNA-seq has promoted new discoveries in nephrology, including diabetic kidney disease^52^, lupus nephritis^53–55^, rejection^56^ and renal tumor^57^. Most of the use of scRNA-seq has focused on intrarenal immune cells but less on regulated necrosis in kidney cells after I/R injury. Pyroptosis was first found in macrophages^58^, and later has also shown in lympocytes^59^, neutrophil^60^ and TECs^40,61^ which was consistent with our results. Pyroptosis-related genes were observed in all clusters of renal cells. Necroptosis in macrophages has been well studied in bacterial infection, atherosclerosis and arthritis^62–64^, but less in kidney diseases^47,65,66^. In this study, we found that necroptosis-related genes were expressed more in macrophages than in TECs; thus, macrophage necroptosis should be more important in the progression of kidney diseases and deserves attention. Studies have also shown that necroptosis and pyroptosis in TECs promote ischemic kidney injury^18,40,66,67^. In our study, we found that genes related to ferroptosis, necroptosis and pyroptosis were regulated in TECs, but the number of TECs expressing ferroptosis-associated genes were more than the other two regulated necrosis. These data suggest that for TECs, ferroptosis might be the most vital form of regulated necrosis following ischemic injury.

There are some limitations in this study: (1) The sample size of human was not big enough to provide sufficient information for assessing the connection between ferroptosis and clinical manifestations. In previous reports, blood hemoglobin and platelet counts were observed to be changed after ferroptosis^68^. Thus, large prospective studies in patients with acute kidney tubular injury are expected. (2) More specific and detailed TECs were not classified in the scRNA-Seq analysis, which did not allow for further sub-cluster analysis of regulated necrosis. It should also be admitted that the changes in RNA expression, such as ACSL4, cannot be used as the definitive markers for execution of ferroptosis and more detailed analyses of regulated necrosis pathways in different kidney cells needs to be explored by the development of proteomics. (3) As we did not detect enough XJB-5-131 in liver homogenate, the underlying mechanism needs to be unveiled, and whether it can be applied in liver diseases also needs to be investigated.

In conclusion, despite these limitations, our study provided evidences of ferroptosis in human kidney diseases. We confirmed the expression of proferroptotic gene ACSL4 in TECs was associated with the severity of kidney function decline. In addition, in this study, we identified a more stable ferroptosis inhibitor, XJB-5-131, which has an affinity to kidney and TECs, could attenuate kidney injury and inhibit lipid peroxidation after I/R injury. By scRNA-seq, we confirmed an important role of ferroptosis in TECs after ischemic injury. Pharmacological targeting of ferroptosis might be an effective way to treat ischemic renal injury, and XJB-5-131 provides a potential and novel therapeutic strategy for renal diseases.

## Methods

### Animals treatment

Male C57BL/6 mice (8−10 weeks old and weighing 20−25 g) were purchased from HuaFukang Company (Beijing, China). Animals were given free access to water and food and provided a 12/12 h light/dark cycle at Tongji Medical School. All experimental procedures were approved and conducted in accordance with the institutional guidelines for animal care.

#### Renal I/R model

After a minimum of 7 days of acclimation, the mice were anesthetized with a 1% sodium pentobarbital solution (6 mL/kg) by intraperitoneal injection and then placed in a prone position to maintain their body temperature at 36.8–37.2 °C during surgery. After a unilateral dorsal incision to expose the kidneys, the renal artery was occluded using a microvascular clamp (RoBoz Surgical Instrument Co, Gaithersburg, MD, USA) for 30 min. After that, the clamps were removed, and the animals were allowed to recover with free access to food and water.

To study the effects of XJB-5-131 on I/R injury, the mice were randomly allocated into the Vehi group and XJB group. The mice were injected intraperitoneally (i.p.) with XJB-5-131 (10 mg/kg) or the respective control 30 min prior to ischemia and for 3 consecutive days after surgery.

### Transmission electron microscopy

The renal cortices were cut into small pieces (1 mm^3^) and fixed with 2.5% glutaraldehyde. Then the samples were dehydrated and embedded. Ultra-thin sections were stained and observed with a transmission electron microscope (Tecnai G^2^ 20 TWIN, FEI, USA).

### Mouse kidney procurement and isolation

The mice were anesthetized and perfused with phosphate buffer saline (PBS). The kidney capsule was removed and digested with gentleMACS and a multi-tissue dissociation kit for 30 min. The digested tissue was passed through a 75-μm cell strainer, and dead cells were removed using a Zombie Aqua™ fixable viability kit (BD Biosciences). Because of the genetic stability of the mice, we obtained two samples of single-cell suspensions from the entire kidneys of five sham mice and five I/R mice by mixing each together for the 10× Genomics studies and sequencing.

### 10× Genomics

10× Chromium single-cell libraries were prepared according to the standard protocol outlined in the manual. Briefly, sorted single-cell suspensions and then single cells were captured by 10 × 3′ barcoded gel beads and oil to generate gel bead-in-emulsions (GEMs). Next, the GEMs were incubated to generate full-length cDNA libraries. Single-cell cDNA libraries were pooled together, cleaned and preamplified by PCR to generate a sufficient mass for sequencing library construction. Sequencing libraries were constructed by following the steps of cDNA fragmentation, end repair and A-tailing, size selection by select beads, adaptor ligation, sample index PCR amplification, and repeat select bead size selection.

### Data preprocessing

The samples were sequenced using a Novaseq 6000. Preliminary sequencing results were converted to FASTQ files with Cell Ranger. Cell Ranger was used for preliminary data analysis and generated a folder with a barcode table, gene table and gene expression matrix. In the sham sample, a total of 8732 cells were isolated and sequenced with a mean of 39,310 reads and a median of 1849 genes detected per cell. In the I/R sample, a total of 10431 cells were estimated with a mean of 27,920 reads and a median of 1859 genes detected per cell.

### Single-cell data analysis

A further downstream analysis was performed using Seurat 3.0 in the R Statistical language. Quality control (QC) was performed with the following parameters, similar to what has been previously reported^52,54,56^: (1) genes expressed less in three cells and cells with less than 200 expressed genes were excluded; (2) cells with more than 30% mitochondrial gene expression were removed. After the QC process, a total of 13,650 cells (6069 sham and 7581 I/R) from two independent experiments were stained for the subsequent analysis and there was a median of 2369 genes per cell. We selected 2000 highly variable genes for cell clustering.

#### In vivo-based pharmacokinetic model

Mice were injected with 3 mg/kg XJB-5-131 and were sacrificed at 0.25, 0.5, 1, 2, 4 and 6 h after injection. Plasma, kidney, liver and brain were collected to assess the XJB-5-131 concentration by LC-MS/MS. For plasma samples, an aliquot of 8 µL was protein precipitated with 160 µL IS, and the mixture was vortex-mixed and centrifuged at 12,000 × *g* for 15 min at 4 °C. Seventy microliters of supernatant was transferred to a 96-well plate and centrifuged for 5 min at 3220 × *g* and 4 °C, and then 6 µL of supernatant was directly injected for LC-MS/MS analysis. For tissue samples, tissue homogenates (kidney, brain and liver) were prepared by homogenizing tissue with threefold volume of homogenization solution (MeOH/15 mM PBS = 1:2). An aliquot of 20 µL was protein precipitated with 400 µL IS, and the mixture was vortex-mixed and centrifuged at 12,000 rpm for 15 min at 4 °C. Seventy microliters of supernatant was transferred to a 96-well plate and centrifuged for 5 min at 3220 × *g* and 4 °C, and then 6 µL of supernatant was directly injected for LC-MS/MS analysis.

### Histological analysis

Paraffin-embedded renal sections (4 μm) were subjected to PAS staining to detect morphological changes in the cortex and medulla, and were scored in a blinded fashion using a semiquantitative design as previously reported^69^.

### Immunofluorescence and immunohistochemistry

Paraffin-embedded renal sections (4 μm) were deparaffinized in xylene, rehydrated in graded alcohol and blocked with 10% goat serum for 30 min. Mouse monoclonal anti-GSDMD (1:50, Santa), mouse monoclonal anti-4-HNE (1:100, R&D), rabbit polyclonal anti-MLKL, goat polyclonal anti-KIM-1 (1:800, R&D), rabbit polyclonal anti-Ki67 (1:100, Abcam), rabbit polyclonal anti-ACSL4 (1:200, Abcam), rabbit polyclonal anti-CD3 (1:100, Dako), rabbit polyclonal anti-ly6G (1:100, Abcam), rat polyclonal anti-F4/80 (1:50, Abcam), and rabbit polyclonal anti-Sox9 (1:100, Abcam) primary antibodies were incubated at 4 °C overnight and subsequently visualized with HRP-conjugated secondary antibody for IHC and fluorescently labeled secondary antibodies for IF. Staining was carefully quantified in each slide by capturing 15 randomly chosen fields in a blind manner, and the data were analyzed using Image Pro Plus software (Media Cybernetics, USA).

### GSH-Px

The levels of GSH-Px in kidneys were determined using GSH-Px assay kit, respectively (Beyotime China) in line with the manufacturer’s instructions.

### MDA assay

Quantification of MDA levels in kidney tissue after I/R or sham operation was performed by using an MDA assay kit (Beyotime).

### Western blot analysis

Renal tissues were lysed in RIPA lysis buffer containing protease inhibitor. Equal amounts of proteins (50 μg) were loaded and separated by 10% SDS-PAGE. The proteins were transferred onto polyvinylidene fluoride membranes (Millipore, USA). The membranes were blocked with 5% skimmed milk in Tris Buffered saline Tween (TBST) for 1 h at room temperature and were then incubated with appropriate primary antibodies against GPX4 (1:10000, Abcam), ACSL4 (1:10000, Abcam, UK), MLKL (1:1000, Abgent), p-MLKL (1:1000, Abcam), RIPK3 (1:1000, ABclonal), p-RIPK3 (1:1000, Abcam), GSDMD (1:1000, Santa) and GAPDH (1:5000, ABclonal) at 4 °C overnight. The blots were washed with TBST for 5 min and incubated with HRP-conjugated secondary antibodies (1:5000; ABclonal) for 1 h. After washing with TBST, the blots were visualized by the enhanced chemiluminescence method (ECL, Bio-Rad). The relative intensity of the target band was normalized to the corresponding loading control intensity and quantified by using ImageJ (NIH, USA).

### Quantitative RT-PCR

Total RNA was extracted from kidney tissue using TRIzol reagent (Takara, Japan). Real-time PCR was carried out using a LightCycler 480 system (Roche, Pleasanton, CA, USA). Relative mRNA for RANTES, MCP-1, IL-6 and GAPDH was quantified in triplicate by SYBR Green RT-PCR. The primers used are listed in Table 3.

**Table 3:** The primer sequences used in the real-time PCR experiment.

Gene name	Forward	Reverse
RANTES	5′-TGCTTTGCCTACCTCTCCCT-3′	5′-AGCACTTGCTGCTGGTGTAG-3′
MCP-1	5′-TAAAAACCTGGATCGGAACCAAA-3′	5′-GCATTAGCTTCAGATTTACGGGT-3′
IL-6	5′-AAGTGCATCATCGTTGTTCATACA-3′	5′-GAGGATACCACTCCCAACAGACC-3′
GAPDH	5′-AGGTCGGTGTGAACGGATTTG-3′	5′-GGGGTCGTTGATGGCAACA-3′

Relative mRNA expression levels were quantified according to the 2^−ΔΔCt^ method and were normalized to the expression levels of GAPDH.

### Statistical analyses

For scRNA-seq and human kidney biopsy experiments, significant differences between the two groups of TECs (Sham TECs and IR TECs) and ACSL4 (ACSL4^High^ and ACSL4^Low^) were analyzed using nonparametric statistics by SPSS 13.0. Clinical parameters are expressed as medians (25th percentiles−75th percentiles) or percentages. Statistical differences between groups were analyzed using nonparametric statistics by SPSS 23.

For mouse experiments, the results are presented as the means ± SEM. Statistical differences between the two groups were analyzed using the unpaired *t* test by GraphPad Prism 6.0. The statistical significance of differences was set at *p* < 0.05 (*p < 0.05; **p < 0.01; and ***p < 0.001).

### Sample collection of human kidney biopsies

This study was carried out in accordance with the Declaration of Helsinki and was approved by the Committee on Research Ethics of Tongji Hospital, Huazhong University of Science and Technology. Seventy Chinese patients in Tongji Hospital were enrolled in this study. Clinical and laboratory information were recorded at the time of the renal biopsy. All patients gave informed written consent.

## Supplementary information

supplementary figure legend

SUPPLEMENTAL figure1

SUPPLEMENTAL figure2

SUPPLEMENTAL figure3
