# Supplementary material for: XJB-5-131 inhibited ferroptosis in tubular epithelial cells after ischemia−reperfusion injury
Source: Cell Death Dis. 2020 Aug 14;11(8):629. doi: 10.1038/s41419-020-02871-6 (PMC7429848; doi:10.1038/s41419-020-02871-6)
Supplement: Supplementary file 1 — supplementary figure legend [file 41419_2020_2871_MOESM1_ESM.docx]

**Supplementary Figure1**. XJB-5-131 attenuated ischemia injured kidney in a dose-independent manner. PAS staining showed a mass of tubular dilutions, casts and loss of tubular brush border in I/R+Vehicle group. XJB-5-131 ameliorated kidney injuries and reduced casts at day 3 after I/R. At dosage of 10mg/kg, XJB-5-131 treatment showed better beneficial than other doses of XJB-5-131 (2.5mg/kg or 20mg/kg). N=5/group. Scale bars =50 μm. Data were presented as the means ± SEM. **p*<0.05, ***p*<0.01, ****p*<0.001.

**Supplementary Figure2.** (A) Scheme of experimental design using sham and I/R kidney tissue samples. (B) Heat map of the selected marker genes in kidney cells to classify the cell clusters. The selected marker genes for each cluster were based on the published data from single-cell analysis in kidneys.

**Supplementary Figure3.** Representative sections of GSDMD immunofluorescence staining from mice kidneys after unilateral I/R at day 5. The white dotted line indicated the renal tubule. The white arrow indicated representative GSDMD distribution in renal interstitial cells, not in renal tubules. Scale bars =50 μm.
