# Supplementary figures and images for: XJB-5-131 inhibited ferroptosis in tubular epithelial cells after ischemia−reperfusion injury
Source: Cell Death Dis. 2020 Aug 14;11(8):629. doi: 10.1038/s41419-020-02871-6 (PMC7429848; doi:10.1038/s41419-020-02871-6)

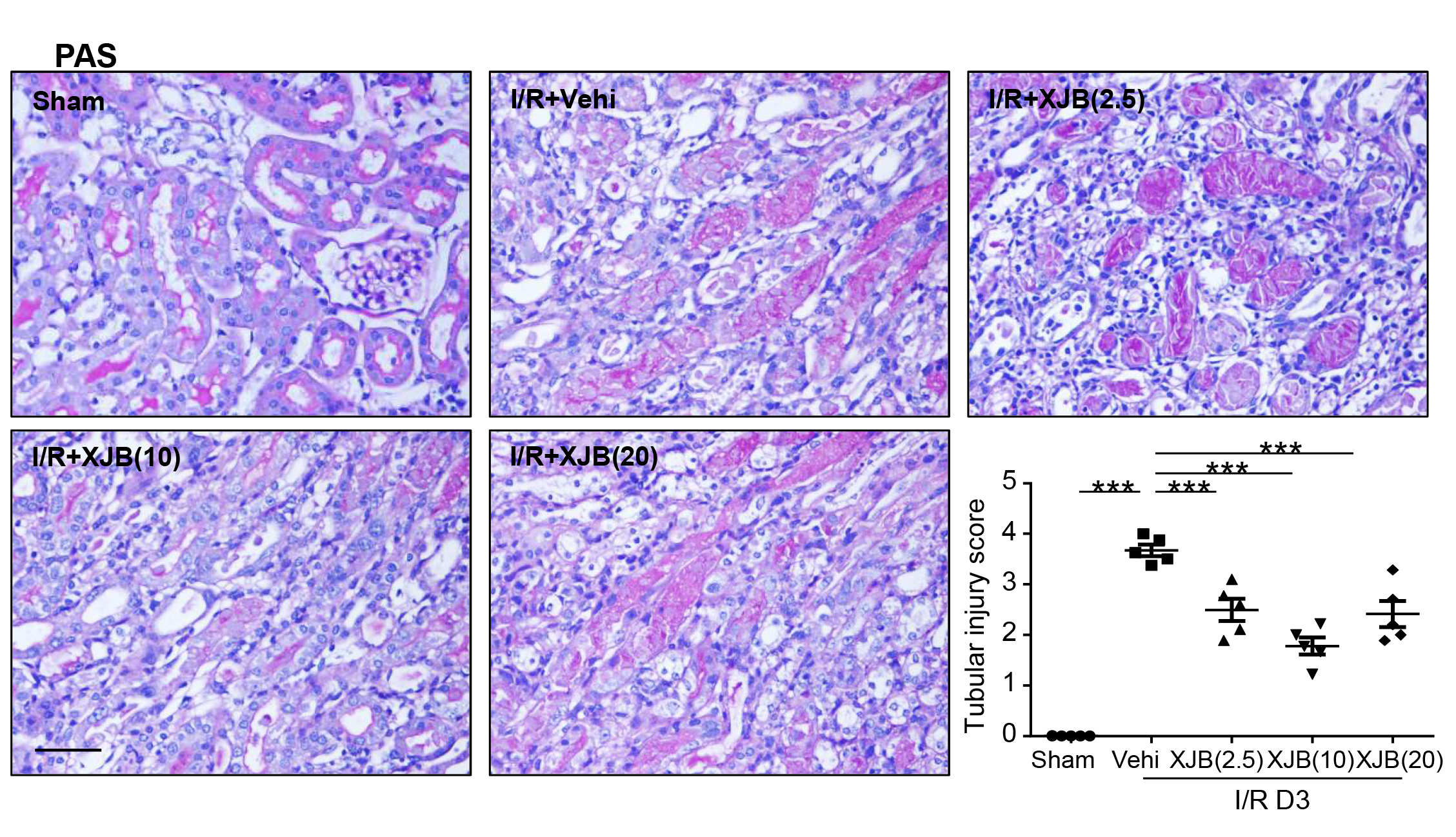

Supplement: Supplementary file 2 — SUPPLEMENTAL figure1 [file 41419_2020_2871_MOESM2_ESM.tif]

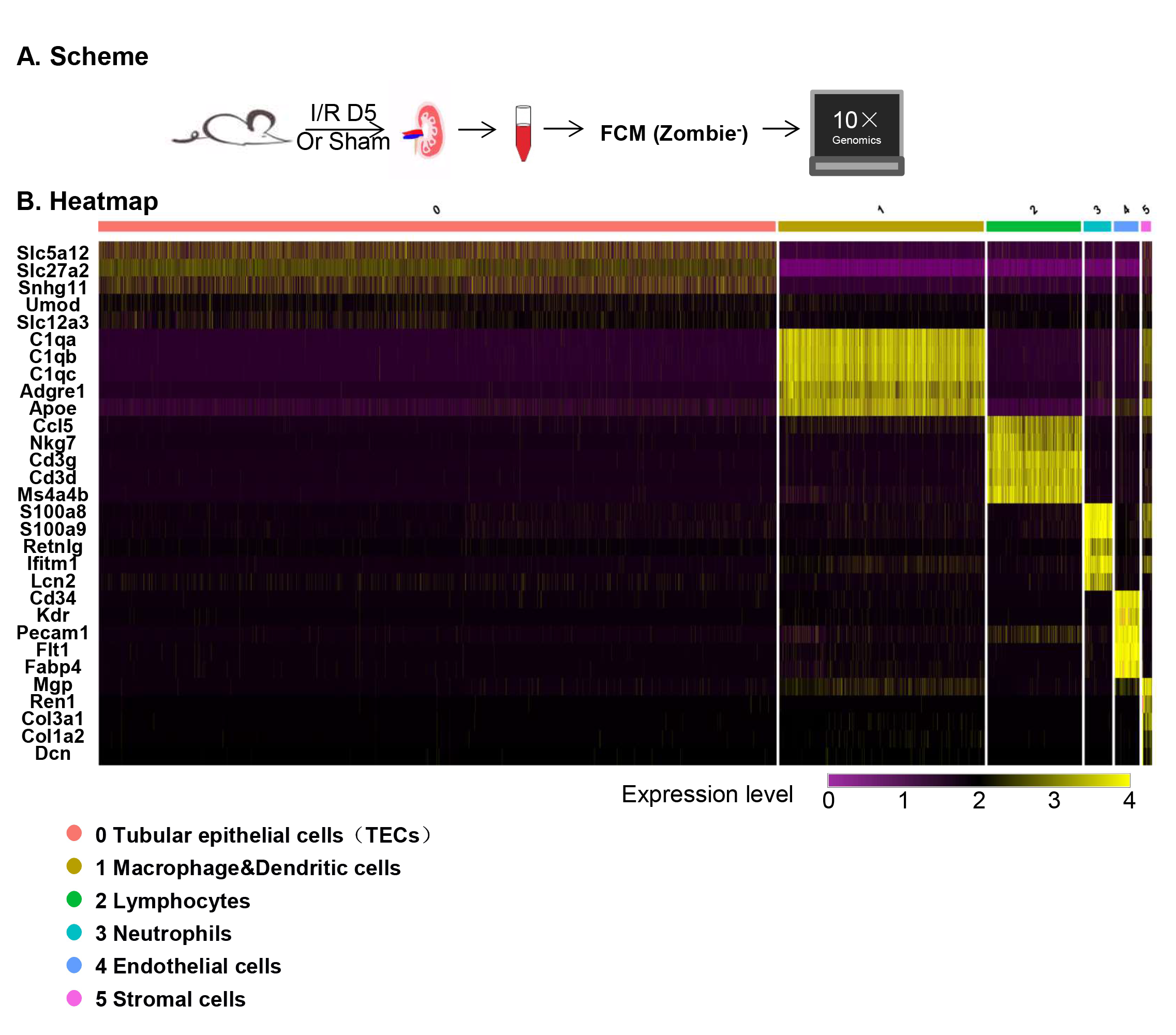

Supplement: Supplementary file 3 — SUPPLEMENTAL figure2 [file 41419_2020_2871_MOESM3_ESM.tif]

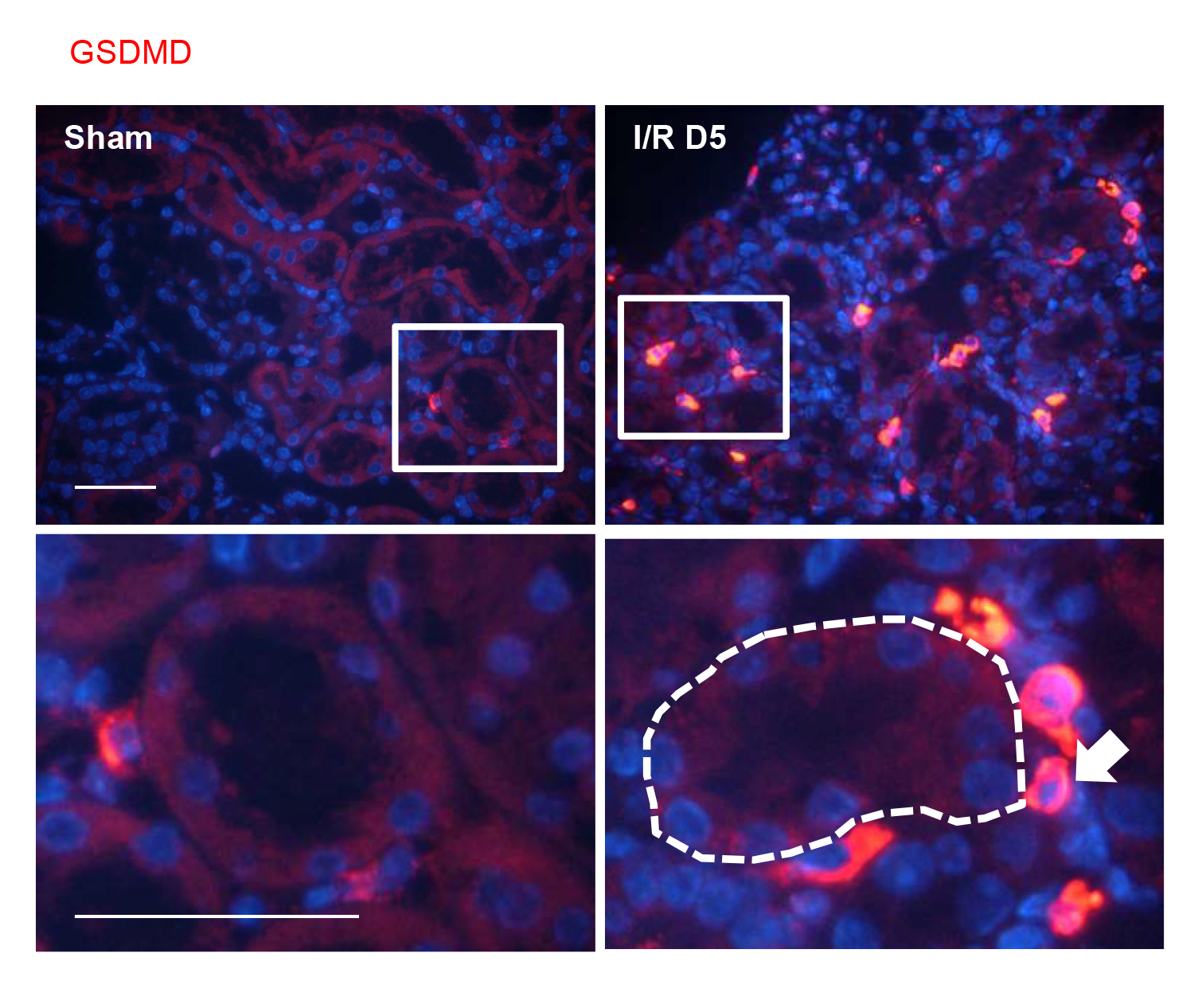

Supplement: Supplementary file 4 — SUPPLEMENTAL figure3 [file 41419_2020_2871_MOESM4_ESM.tif]
